# Approaching the Families of Potential Deceased Organ Donors: An Overview of Regulations and Practices in Council of Europe Member States

**DOI:** 10.3389/ti.2023.11498

**Published:** 2023-09-12

**Authors:** Sándor Mihály, Anikó Smudla, Beatriz Dominguez-Gil, Alicia Pérez, Francesco Procaccio, Emanuele Cozzi, Marta López Fraga, Danica Avsec, Axel Rahmel, John Forsythe, Franz Immer, Janis Jushinskis, Alex Manara

**Affiliations:** ^1^ Hungarian National Blood Transfusion Service, Budapest, Hungary; ^2^ Department of Anaesthesiology and Intensive Therapy, Faculty of Medicine, Semmelweis University, Budapest, Hungary; ^3^ Organización Nacional de Trasplantes, Madrid, Spain; ^4^ National Transplant Centre, Rome, Italy; ^5^ Thoracic Surgery Unit, Department of Cardiac, Thoracic and Vascular Sciences, School of Medicine and Surgery, University of Padua, Padua, Italy; ^6^ European Directorate for the Quality of Medicines and HealthCare (EDQM), Strasbourg, France; ^7^ Institute for Transplantation of Organs and Tissues of the Republic of Slovenia, Slovenija-Transplant, Ljubljana, Slovenia; ^8^ Deutsche Stiftung Organtransplantation, Frankfurt am Main, Germany; ^9^ NHS Blood and Transplant, Bristol, United Kingdom; ^10^ Swisstransplant, National Foundation for Organ Donation and Transplantation, Bern, Switzerland; ^11^ Latvian Transplantation Centre, Riga, Latvia; ^12^ The Intensive Care Unit, Southmead Hospital, North Bristol NHS Trust, Bristol, United Kingdom

**Keywords:** deceased organ donation, family approach, family communication, consent for organ donation, Council of Europe

## Abstract

The primary aim of this study was to describe regulations and practices concerning the family approach to discuss donation, specifically after the neurological determination of death, one of the most challenging steps in the donation pathway. A secondary objective was to assess the impact of legislation on consent rates for organ donation. The Council of Europe surveyed 39 member states about national regulations, practices, and consent rates; 34 replied. Opt-out legislation is present in 19, opt-in in 9 and a mixed system in six countries. An opt-out register is kept by 24 countries and an opt-in register by 18 countries, some keeping both. The mean consent rate was 81.2% of all family approaches. Most countries regulate how death using neurological criteria is confirmed (85.3%), while regulation of other aspects of the deceased donation pathway varies: the timing of informing the family about brain death (47.1%) and organ donation (58.8%), the profile of professional who discusses both topics with the family (52.9% and 64.7%, respectively) and the withdrawal of treatment after brain death (47.1%). We also noted a mismatch between what regulations state and what is done in practice in most countries. We suggest possible reasons for this disparity.

## Graphical Abstract

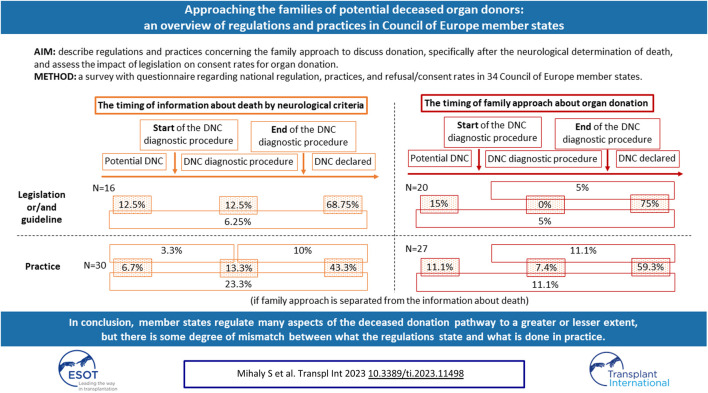

## Introduction

Organ transplantation is often the only treatment option for patients with end-stage organ failure but is limited by the availability of organs [1]. To maximize the availability of organs, all potential organ donors must be identified, referred, and managed along pathways that ensure most potential donors become actual donors. The ultimate objective is for all nations to achieve self-sufficiency in transplantation, as recommended by the Madrid Resolution [2].

Families declining organ donation is an important reason for the loss of donation potential, the rates of which vary among countries but remain a matter of concern in Europe. Consent to organ donation is influenced by many factors, particularly a known donation decision made by the deceased during life and whether a trained individual is involved in the family conversation [3]. The impact of legislation is less clear. In 2017, 19 Council of Europe countries that had implemented opt-out legislation achieved 27.4 deceased donors per million population (pmp), more than twice that achieved in the 11 countries with opt-in legislation (12.1 pmp). Interestingly, the average family decline rate in states with opt-in legislation (15.8 pmp in 6 countries) was double that in states with opt-out systems (7.3 pmp in 13 countries). The family decline rate in opt-in countries exceeded the deceased donor rate (15.8 vs. 12.1 pmp). In opt-out countries the decline rate was a quarter that of deceased donors (7.3 pmp vs. 27.4 pmp). The number of families declining donation as a proportion of all family donation conversations (decline rate) was 20.4% in 13 opt-out countries, compared with 47.8% in 6 opt-in countries [4]. However, the decline rate may be calculated differently, and legislation is not necessarily the most important influencing factor. Public support for donation and transplantation, trust in the individual jurisdiction’s system, spiritual or cultural beliefs and practices for approaching families to discuss donation may have a greater impact [5].

A decline to organ donation can represent an individual’s decision expressed during life by registering an opt-out decision, but often results from a decision made on the potential donor’s behalf by their family [6]. Consent to organ donation is also influenced by factors associated with the family approach: when, how, and by whom the family is informed about donation opportunities [7]. The timing and temporal separation (“decoupling”) of discussions regarding brain death or a decision to withdraw life-sustaining treatments from discussions seeking family support for organ donation can also influence consent [3, 8, 9]. Some countries have regulations (legislation or guidance) on how professionals should approach families to discuss deceased donation [10–16]. However, to the best of our knowledge, no granular information is available on the regulatory frameworks and current practices concerning the family approach in individual member states.

The main objective of this study is to describe current regulations and practices covering the family approach to discuss donation, specifically for DBD (donation after brain death/neurological determination of death). A secondary objective is to assess and describe the impact of legislation on consent rates for organ donation. This may be useful for individual countries reviewing their current regulatory framework and its practical implementation.

## Materials and Methods

The European Committee on Organ Transplantation of the Council of Europe (CD-P-TO) had accepted a project proposal, then established an *ad hoc* working group who held a consensus meeting to design a questionnaire that was endorsed by the Committee. The questionnaire consisted of 33 questions eliciting 56 responses on the following areas: the regulations and practices regarding discussing a diagnosis of brain death with families, the approach for organ donation, and the family decline/consent rate, questions regarding donation after the circulatory determination of death (DCD), and questions on the possibility and regulation of organ donation from non-citizen/non-resident deceased persons. The last two items are not included in this study due to low response rate and an intention to publish separately. The questionnaire was sent to representatives of 39 Council of Europe countries during the second half of 2021 (see Supplementary Material), who completed it using information and validated data obtained from their official national sources. All the data and information were reviewed by the authors who requested further clarification from the respondents during the validation. Other donation metrics for 2016 to 2020 were derived from the *Newsletter Transplant* [4, 17–20].

Data were analyzed with the Statistical Package for the Social Sciences version 20.0 (SPSS Inc., Chicago, Illinois) including descriptive statistics and tests of significant differences. Continuous variables were analyzed with independent samples *t*-test for variables with two categories. Fisher’s exact test was used for statistical differences between two categorical variables. The significance level was set to 5% (*p* ≤ 0.05).

Definitions (possible, potential, actual, utilized organ donor) used in this paper have been adopted by the authors from the Critical Pathway [1].

## Results

The response rate was 87% (34 of 39 member states who received the questionnaire).

### National Regulations Regarding Brain Death and Consent for Organ Donation

A summary of national regulatory frameworks is shown in Table 1.

**TABLE 1:** Legislation on consent in the Council of Europe member states.

	Country	Organ and tissue donation consent models	Family veto in opt-out systems	Family veto in opt-in systems	Opt-out registry	Opt-in registry
1	Andorra	Presumed consent (opt-out)	No	Yes	No	No
2	Austria	Presumed consent (opt-out)	No	—	Yes	No
3	Belarus	Presumed consent (opt-out)	No	—	Yes	No
4	Belgium	Presumed consent (opt-out)	No	—	Yes	Yes
5	Bulgaria	Presumed consent (opt-out)	No	—	Yes	No
6	Croatia	Presumed consent (opt-out)	No	—	No	No
7	Cyprus	Other	—	Yes	Yes	Yes
8	Czech Republic	Presumed consent (opt-out)	Yes	—	Yes	No
9	Denmark	Informed/explicit consent (opt-in)	—	Yes	Yes	Yes
10	Estonia	Presumed consent (opt-out)	No	Yes	Yes	Yes
11	Finland	Presumed consent (opt-out)	No	—	No	No
12	France	Presumed consent (opt-out)	No	—	Yes	No
13	Georgia	Informed/explicit consent (opt-in)	—	No	No	Yes
14	Germany	Informed/explicit consent (opt-in)	—	No	No	No
15	Greece	Informed/explicit consent (opt-in)	—	Yes	Yes	Yes
16	Hungary	Presumed consent (opt-out)	Yes	—	Yes	No
17	Ireland	Informed/explicit consent (opt-in)	—	No	No	No
18	Israel	Informed/explicit consent (opt-in)	—	No	No	Yes
19	Italy	Other	No	Yes	Yes	Yes
20	Latvia	Presumed consent (opt-out)	No	—	Yes	Yes
21	Lithuania	Informed/explicit consent (opt-in)	—	Yes	No	Yes
22	Moldova	Other	No	Yes	Yes	Yes
23	Netherlands	Presumed consent (opt-out)	No	No	Yes	Yes
24	Norway	Presumed consent (opt-out)	No	Yes	No	No
25	Poland	Presumed consent (opt-out)	No	—	Yes	No
26	Portugal	Presumed consent (opt-out)	Yes	—	Yes	No
27	Romania	Informed/explicit consent (opt-in)	—	No	No	Yes
28	Serbia	Presumed consent (opt-out)	No	—	Yes	No
29	Slovak Republic	Presumed consent (opt-out)	No	—	Yes	No
30	Slovenia	Other	No	Yes	Yes	Yes
31	Spain	Presumed consent (opt-out)	No	Yes	Yes	Yes
32	Sweden	Other	No	—	Yes	Yes
33	Switzerland	Informed/explicit consent (opt-in)	—	Yes	Yes	Yes
34	United Kingdom	Other	No	No	Yes	Yes

**FIGURE 1:**
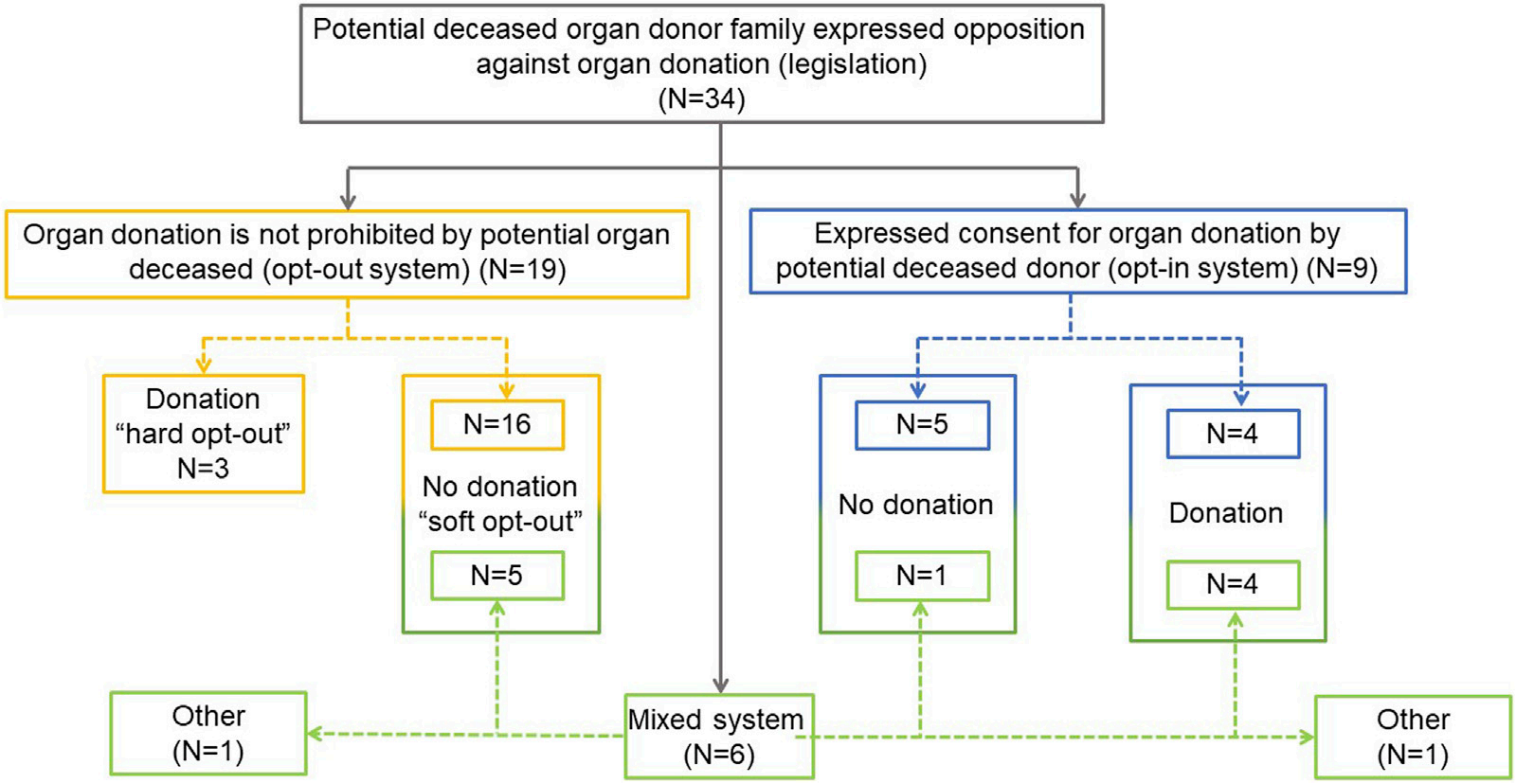
National legislation applicable to the family approach and consent for organ donation.

Opt-out (presumed consent) legislation is present in 19 countries (56%), opt-in legislation in 9 (26%) and a mixed legal system (countries without a defined opt-out or opt-in model) in the remaining 6 (18%). Twenty-one countries (16 opt-out and 5 mixed) operate a system where donation will not proceed if the family objects, even if there is no written objection from the donor. Three countries operate a “hard opt-out” system: donation will proceed despite family opposition, unless there is written evidence that the deceased chose not to be an organ donor. In 6 (5 opt-in and 1 mixed) countries families can override an opt-in decision and donation will not proceed. Finally, in 11 countries (3 “hard opt-out,” 4 opt-in and 4 mixed system countries) organ donation will proceed despite family opposition when there is written evidence of the deceased’s decision to donate (Figure 1).

An opt-out register is available in 24 countries (71%): 15 opt-out, 3 opt-in and 6 mixed system countries. Four countries with opt-out legislation have no opt-out register. An opt-in register is available in 18 countries (53%), including 5 opt-out and all 6 mixed system countries. Among the 16 countries with no opt-in register, 2 require informed/explicit consent (Table 1).

The determination of death using neurological criteria (DNC) is regulated by legislation in 25 (73.5%) countries and by guidelines only in 5 countries (14.7%). Four countries have both.

**FIGURE 2:**
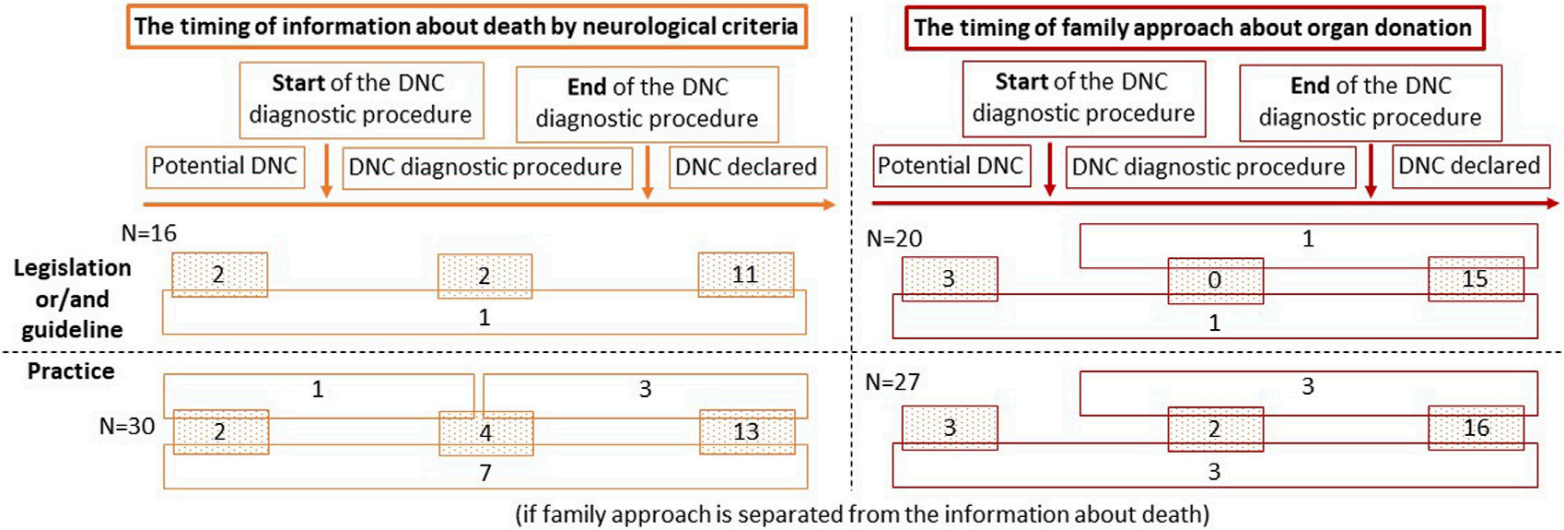
The timing of information given to the family about death by neurological criteria and the timing of family approach to address the possibility of organ donation. Abbreviations: DNC, death by neurological criteria.

The time at which the family is informed about a brain death diagnosis is regulated in 16 countries (47.1%): 6 by legislation, another 6 by guidelines, and by a combination of both in 4 countries. In 3 (2 + 1) of these countries, the family may be informed that the patient’s condition may progress to brain death before DNC is confirmed. However, in 11 of 16 countries the family can only be informed about DNC after the diagnosis is confirmed. In 3 (2 + 1) countries, the family may be informed that the clinical condition is compatible with DNC before the diagnosis is confirmed (Figure 2).

In 20 countries (58.8%) the timing of the family approach to discuss donation is regulated: by legislation in 6 countries and by guidelines in 9; 5 countries have both. Organ donation can be discussed when DNC is a likely outcome but has not yet occurred (3 + 1 countries; *n* = 4; 20%), when the patient has a clinical condition consistent with DNC, but before the diagnosis is confirmed (1 + 1 countries; *n* = 2; 10%), or only after DNC has been officially declared (15 + 1+1 countries; *n* = 17; 85%) (Figure 2).

**FIGURE 3:**
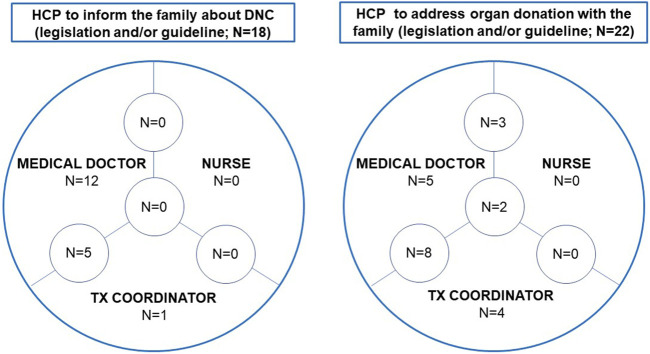
The healthcare professional to inform the family about death by neurological criteria and to address organ donation with the family. Abbreviations: DNC, death by neurological criteria; HCP, healthcare professional.

The healthcare professional (HCP) who should inform the family about DNC is regulated in 18 countries (52.9%): by legislation in 9 (26.5%), by a guideline in 6 (17.7%) and by a combination of both in 3 (8.8%). The HCP should be a medical doctor (*n* = 17; 94.4%) or a donor coordinator (who can be a medical doctor) (*n* = 6; 33.3%). Both types of professionals are permitted in 5 countries (27.8%) (Figure 3).

The HCP who discusses organ donation with the family is regulated in 22 countries (64.7%): by law in 12, by a guideline in 6, and by a combination in 4 countries. A medical doctor is required to do this task in 18 countries (81.8%), a nurse in 5 (22.7%) and the donor coordinator in 14 (63.6%). All 3 HCPs can approach families to discuss organ donation in 2 countries (9.1%), medical doctors and nurses in 3 countries (13.3%) and medical doctors and donor coordinators in a further 8 countries (36.4%) (Figure 3).

The information that should be provided to the family is detailed in the regulatory framework of 17 countries (50%): guidelines in 13 countries, legislation in 2 and both in 2 countries.

Finally, the withdrawal of mechanical ventilation after confirming DNC is regulated in 16 countries (47.1%) when organ donation cannot proceed: by legislation in 12 and by guidelines in 4. It is also regulated in 18 countries (52.9%) when a family declines the offer of organ donation after a diagnosis of DNC: by legislation in 13 countries and by guidelines in 5.

### Practices Regarding the Family Approach for Potential Organ Donation

There was no donation activity over the survey period in 2 participating countries; therefore, practices were surveyed in the other 32 countries.

Although in two countries HCPs (one country with legislation and one country without legislation or guideline) may deliver information on organ donation in one step, a gradual approach is used in most countries (56.3%), as many families need time to process and accept the death of their loved one before making a decision about organ donation. Decoupling the conversation about a brain death diagnosis from the approach for organ donation is used in 25% of the countries.

In most countries (13 + 3+7 countries; *n* = 23; 71.9%) the family is usually informed about DNC when the diagnosis has been officially declared. Less commonly (4 + 1+3 + 7 countries; *n* = 15; 46.9%) the family is informed that the patient’s clinical condition is consistent with DNC before the diagnosis has been confirmed. In 10 countries (1 + 2+7 countries; 31.3%) DNC is communicated to the relatives at an early stage, when it is a likely outcome but has not yet occurred (Figure 2). In countries where the time to inform families about DNC is not regulated, the family is more commonly informed when the patient has a clinical condition consistent with DNC (56.3% vs. 37.5%) or when DNC has been officially declared (87.5% vs. 56.3%) when compared to countries with legislation and/or guidelines. In those countries where there is legislation to inform the family about DNC after it has been confirmed, in practice this is done on 83.3% of occasions. By contrast, only two-thirds of countries that use guidelines will follow them in practice. In these countries, the possibility of DNC is discussed with the family at an early stage, when it is a likely outcome but has not yet occurred. Although these early discussions are not permitted by legislation and/or guidelines, they may still occur in 3 of 16 (18.8%) countries (Table 2).

**TABLE 2:** National legislation and guidelines regarding informing the family about diagnosing death using neurological criteria and what happens in practice.

Legislation and guidelines			What happens in practice		
			HCP may inform the family about DNC when DNC is expected in the short term	HCP may inform the family about DNC when DNC is suspected but not yet confirmed	HCP informs the family about DNC only after DNC has been confirmed
			Yes N (%)	Yes N (%)	Yes N (%)
Does the legislation/guideline specify the HCP responsible for informing on DNC?	Yes	16	5 (31.3)	6 (37.5)	9 (56.3)
	No	16	5 (31.3)	9 (56.3)	14 (87.5)
If yes, specify the type of regulation	Legislation	6	0 (0)	1 (16.9)	5 (83.3)
	Guideline	6	2 (33.3)	2 (33.3)	4 (66.7)
	Legislation + Guideline	4	1 (21)	0 (0)	3 (75)
According to the legislation/guidelines:					
The HCP can inform the family about DNC when it has not yet occurred but is expected to do so in the short term	Yes	3	3 (100)	1 (33.3)	0 (0)
	No	13	2 (15.4)	5 (38.5)	9 (69.2)
The HCP can inform the family about DNC when the diagnosis is suspected but has not yet been confirmed	Yes	3	1 (33.3)	1 (33.3)	1 (33.3)
	No	13	4 (30.8)	5 (38.5)	8 (61.5)
The HCP can only inform the family after DNC has been confirmed	Yes	12	3 (21)	4 (33.3)	8 (66.7)
	No	4	2 (50)	2 (50)	1 (25)

Abbreviations: DNC, death using neurological criteria; HCP, healthcare professional.

In 22 of 32 countries (16 + 3+3 countries; 68.8%), the option of organ donation is only discussed with family after DNC has been confirmed, the family has been informed of the diagnosis and given time to accept that their relative has died. A less frequent practice is to discuss organ donation with the family earlier when the patient has a clinical condition consistent with DNC, but before the diagnosis (2+3+3 countries; 25%) or when progression to DNC is likely (3+ 3 countries; 18.8%) (Figure 2). Relatives are approached for organ donation after the confirmation of DNC in 100% of countries without legislation and/or guidelines on the timing of the family approach, and in 57.9% of countries with any type of regulation. However, the implemented practice is different; families are approached after confirmation of DNC in 80% of the countries with legislation and in 44.4% of those with guidelines. Only 50% of countries follow this practice even if it is regulated by both legislation and guidelines. In 15 countries, discussion about the possibility of organ donation is prohibited before DNC has been confirmed. Despite the legislation and/or guidelines, in practice this happens in 3 countries (Table 3).

**TABLE 3:** National legislation and guidelines regarding the timing of the family approach for organ donation and what happens in practice.

Legislation and guidelines			What happens in practice		
			HCP may inform the family about organ donation when DNC is expected in the short term	HCP may inform the family about organ donation when DNC is suspected but not yet confirmed	HCP may inform the family about organ donation after DNC has been confirmed
			Yes N (%)	Yes N (%)	Yes N (%)
Does the legislation/guidelines specify when the HCP must/should approach the family to discuss organ donation?	Yes	19	4 (21.1)	5 (26.3)	11 (57.9)
	No	11	2 (18.2)	3 (27.3)	11 (100)
If yes, specify the type of regulation	Legislation	5	1 (20)	1 (20)	4 (80)
	Guideline	9	3 (33.3)	2 (22.2)	4 (44.4)
	Legislation + Guideline	5	0 (0)	2 (33.3)	3 (66.7)
According to the legislation/guidelines:					
The HCP must/should approach the family to discuss organ donation when DNC has not yet occurred but is expected to do so in the short term	Yes	4	3 (75)	2 (50)	1 (25)
	No	15	1 (6.7)	3 (20)	10 (66.7)
The HCP must/should approach the family to discuss organ donation when the diagnosis of DNC is suspected but has not yet been confirmed	Yes	2	1 (50)	1 (50)	0 (0)
	No	17	3 (17.7)	4 (23.5)	11 (64.7)
The HCP must/should approach the family to discuss organ donation only when the diagnosis of DNC has been confirmed	Yes	16	2 (12.5)	3 (18.8)	10 (62.5)
	No	3	2 (66.7)	2 (66.7)	1 (33.3)

Two countries (one with legislation and one without legislation and/or guidelines) did not comment on the practice regarding informing the family about organ donation.

Abbreviations: DNC, death using neurological criteria; HCP, healthcare professional.

The majority (28 countries) prefer decoupling the conversation informing relatives that DNC has been confirmed from the conversation exploring the option of organ donation. The conversations are usually separated in time and may be led by a different HCP. For example, a medical doctor informs and discusses the confirmation of DNC with the family and the same doctor, or a donor coordinator, explores the potential for organ donation with them. In practice, the conversation about the confirmation of DNC is led by a medical doctor in 28 countries and by the donor coordinator in the other 8 countries. In two countries not regulating this process, this conversation can be led by a nurse. In practice donor coordinators lead the conversation regarding the confirmation of DNC in only 3 of 6 countries where this practice is permitted.

Nurses and donor coordinators are, however, more commonly involved in the organ donation conversation with the family. The family is approached by a medical doctor (alone on 15.6% of occasions, with a donor coordinator in 31.3%, and with a nurse in 9.4%), by a donor coordinator alone in 25%, and by all in 15.6% (one country did not answered this question). In eight countries, donor coordinators are not allowed to participate in the family approach to discuss organ donation; despite this, they are involved in the family approach in 2 of these countries.

### Information on Family Decline/Consent Rate for Organ Donation

Our study results and data from the *Newsletter Transplant* [4, 17–20] shows that the annual number of family interviews pmp in the DBD setting between 2016 and 2020 varied among countries (mean 25.0–27.9). In Council of Europe member states there are on average 1.8 times more family approaches for donation than there are actual DBD donors (range 0.97–6.8 times more family approaches).

The mean proportion of the number of family declines to the number of family approaches was 18.8% (SD: 12.8%; *n* = 13). Two countries reported no family declines during the 5 years investigated. The mean family decline rate in comparison with the DBD rate was 30.5% (SD: 22.2%).

In view of the limited responses, the annual data for 2012–2017 from the *Newsletter Transplant* publications of the Council of Europe were also analyzed. In the examined 6 years period, 20 countries reported data for an average of 4.6 years. The rate of family declines was 26.7% as a proportion of the number of conversations (SD: 9.8%).

## Discussion

The World Health Organization’s Guiding Principles on human cell, tissue and organ transplantation establish that “Organs may be removed from the bodies of deceased persons for the purpose of transplantation if: a) any consent required by law is obtained, and b) there is no reason to believe that the deceased person objected to such removal” [2, 22].

Accordingly, donation and transplantation systems worldwide must develop strategies to exclude any known objection to donation by the potential donor. Jurisdictions should also introduce legislation and/or guidance to regulate the consenting process. Consent legislation is rooted in one of four principles: altruism (opt-in and opt-out), incentivizing (financial and non-financial), mandating (the law obliges all adults to register their donation decision), and confiscating (organs considered a public resource). Systems primarily based on altruism are the most common. In opt-in systems, organs can be recovered from a deceased individual if the person or their legally recognized representative expressly consents to it. In opt-out systems, organs may be recovered from a deceased individual, unless they had previously expressed their opposition to donation [23].

Opt-out consent systems are more widespread in Europe and recently more European countries have introduced opt-out legislation. Netherlands and England implemented opt-out legislation in 2020 [24], Scotland in 2021, Northern Ireland plans to implement opt-out legislation in 2023 and Switzerland in2024. Despite this, the evidence that opt-out systems increase consent or donation rates is not scientifically robust and remains inconclusive [21, 25]. An individual’s donation decision should always be established as best as possible and the individual’s autonomy and right to self-determination should be respected. However, in practice families may overrule this principle because some countries’ legislation allows them to do so. Family overrides raise ethical questions in both opt-in and opt-out systems. Some consider that overriding an active decision to opt-in made by an individual during their lifetime breaches that individual’s autonomy. Others may also question the ethics of allowing a family override in opt-out countries, since arguably an individual is more likely to record a strong objection to donation than they are to record a willingness to donate. Overrides also undermine the philosophy of utilitarianism.

Therefore, in many European countries, there is a mismatch between the legislation and the way consent to organ donation is ascertained in practice. The implementation of practices that are not necessarily aligned to the legislation and guidance may occur because HCPs choose to incorporate deeply rooted societal values, etiquettes, and traditions in the way they approach and deal with grieving, bereaved families. Another issue perpetuating this mismatch is that when an individual has not registered a decision to be an organ donor or informed their family of this decision, the default position in practice is to assume that the individual did not wish to be an organ donor. This assumption influences the consent rate in both consent models, significantly reducing the donor pool. These issues are important when training HCPs on how best to approach the family for organ donation, particularly when the potential donor’s decision is unknown.

Some countries with either opt-in or opt-out legislation operate both opt-in and opt-out registers. Other countries do not maintain either type of register, irrespective of whether they have implemented opt-in or opt-out legislation. It is unclear whether registers increase a country’s consent rate or improve other donation metrics. Their impact is also difficult to assess when families are allowed to override an individual’s registered organ donation decision. Opt-in registers are, however, helpful in that the consent rate is significantly increased when the family and HCPs know that the individual had registered a decision to donate their organs, compared to when their decision is unknown [3].

The process of diagnosing DNC is regulated in all member states, with most preferring to use legislation, possibly in the belief that it is stronger and safer than guidance. The timing of delivering information about brain death and organ donation to the family, and who delivers it, is regulated in half of the countries, indicating that these areas of practice are considered important enough to justify regulation and reduce variations in practice. Since the determination of death must not be influenced by any consideration of donation, more than half the responding counties have introduced regulations to allow the withdrawal of mechanical ventilation and organ support after a diagnosis of DNC has been made in situations where organ donation cannot proceed. These regulations help increase the public’s acceptance and understanding that DNC is death, and that all organ support will be stopped. Post-mortem organ donation simply influences the timing of withdrawal of ventilation.

The timing of discussing brain death and the possibility of organ donation with the family is regulated in most countries, and usually involves separate conversations. In practice both conversations take place at an earlier stage than would be allowed by regulation. In countries that do not regulate the timing of these conversations, information about organ donation is usually provided only after the confirmation of brain death.

This practice of only approaching the family after the confirmation of death is, however, only relevant to the practice of DBD. It is not possible in controlled DCD or in the setting of Intensive Care to Facilitate Organ Donation (ICOD). ICOD is the initiation or continuation of intensive care measures with the intention of maintaining donation potential in patients with a devastating brain injury where death is anticipated, and active treatment is deemed futile [26, 27]. The incorporation of organ donation into their end-of-life plan can only be achieved following a discussion with the family before the patient dies, informing them of the purpose of initiating or continuing intensive care and establishing whether this is consistent with the patient’s values and preferences. Different processes are required for these ethically, professionally, and legally challenging pathways, and regulatory frameworks are essential for such pathways to succeed [28].

Member states of the Council of Europe vary as to who should discuss brain death or organ donation with families, and there is a mismatch between the regulations and actual practice. While there is little evidence to support which HCP is best placed to discuss brain death with families, it is reasonable to expect that this is best done by HCPs with knowledge and expertise of brain death and training on how to communicate. Similarly, it is reasonable to expect that those with knowledge and expertise of organ donation and training in discussing organ donation are best placed for this task. There is significant evidence that when trained donor coordinators lead this conversation, the consent rate is significantly higher than when other HCPs do this [3].

Given our secondary objective of assessing and describing the impact of legislation on organ donation consent rates, the results of our “snapshot” should be interpreted with caution before drawing any conclusions. For example, there is wide variability in the relationship between the number of family approaches weighted by population and the number of deceased donors: one country had 7 times more conversations about donation pmp than organ donors pmp. It is easy to conclude that this is due to the timing of the approach to the family or who leads that approach. It is also possible to conclude that this is a result of other regulations or practices in that country. Such conclusions are, however, unjustified as they are narrow in focus and do not consider the wider picture of the different values and traditions held by the country’s population and HCPs.

Data on the number of family declines to organ donation were provided by only 13 of the 34 respondents (38%), so it is impossible to draw any conclusions on the effect of a country’s legislation and its practices on the consent rate for organ donation. It is essential that all countries in the Council of Europe take responsibility for collecting, recording, and sharing data on the number of family approaches for organ donation and whether such conversations result in a family consenting to or declining organ donation.

**FIGURE 4:**
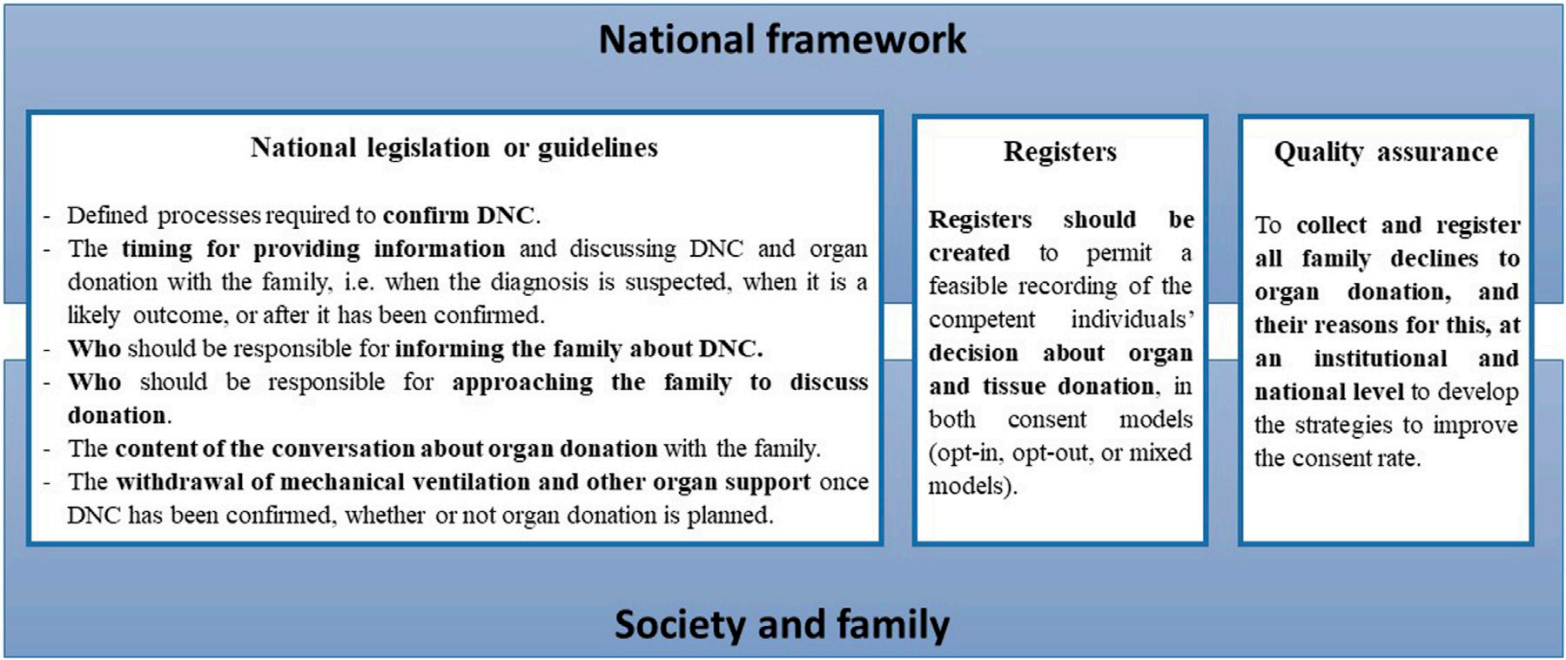
The recommendation to member states of the Council of Europe to improve the quality of the organ donation process. A robust national framework is built to address all the relevant legal, professional, ethical and practical issues associated with deceased organ donation pathways and transplantation. The public is provided updated information on the national legislation about deceased organ donation procedures and donation metrics. This should be responsibility of the relevant national organ donation authority and the government.

The quality of the organ donation process may be improved by our recommendations (Figure 4). More granular data on all aspects of the process of approaching a family for donation will be required if we are to identify and understand the modifiable factors that may influence the outcome of such conversations at a local, national, and international level. It is also important to identify why families who initially decline organ donation later consent to donation, and why some families who initially consent to donation withdraw that consent. Any assessment of whether one consent system is superior to another should consider not only donation metrics, but also other relevant outcomes from the donor family perspective.

In conclusion, the public and HCPs should be made aware of the regulations governing deceased donation in their country and how they are interpreted and implemented in practice. This is particularly true for the consent model established in their jurisdiction. This will allow individuals to consider their donation decision, record it and make their families aware of that decision. Our study shows that many Council of Europe member states regulate many aspects of the deceased donation pathway. Some states use legislation, guidelines, or both to regulate each step of the pathway; other states do not regulate some steps at all. The regulations vary among individual states, but in most states, there is some degree of mismatch between what the regulations state and what is actually done in practice. The reasons for this mismatch need to be better understood. In some situations, it is possible that HCPs are unaware of the regulations. However, it is also possible that the regulations do not align with routine practice. Finally, it is likely that organizations and individuals interpret and implement regulations in a fashion that they believe respects the long-standing traditions and etiquettes of families and of that country, all of which tend to be deeply rooted when dealing with death, bereavement, and grief.

## Data Availability

The original contributions presented in the study are included in the article/Supplementary Material, further inquiries can be directed to the corresponding author.

## Abbreviations

CD-P-TO, European Committee on Organ Transplantation of the Council of Europe; DBD, donation after brain death (neurological determination of death); DCD, donation after the circulatory determination of death; DNC, to determine death using neurological criteria; HCP, healthcare professional; ICU, intensive care unit; PMP, per million population; SD, standard deviation.
