# Supplementary material for: Approaching the Families of Potential Deceased Organ Donors: An Overview of Regulations and Practices in Council of Europe Member States
Source: Transpl Int. 2023 Sep 12;36:11498. doi: 10.3389/ti.2023.11498 (PMC10520243; doi:10.3389/ti.2023.11498)
Supplement: Supplementary file 1 [file DataSheet1.PDF]

TO126 - Family approach during the deceased organ donation process: questionnaire on legislation, practices, and timing

To Council of Europe CD-P-TO national representatives

Dear colleagues,

The aim of this survey is to analyse regulations and practices in Europe regarding the timing and circumstances of family approach during the deceased organ donation process. The survey focuses primarily on donation following neurological determination of death (DNDD), however, there are also dedicated sections on donation after circulatory determination of death (DCDD) and non-resident deceased organ donors at the end of the survey.

Please answer the questions as they relate to the legislation / guidelines / routine practice in your country.

Where a member state has appointed several representatives to the CD-P-TO, please co-ordinate internally so as to submit only one official response per country.

Some of the questions require detailed practical information. Please contact experienced professionals about family approach in your country to provide you with this information, as necessary.

The deadline for submission of responses is **31 August 2021.**

We are convinced the results of this survey will be of great value for health authorities and professionals and will help to identify best practices and areas for improvement across Europe.

We thank you in advance for your co-operation and participation in this initiative.

Transplantation team, EDQM

There are 56 questions in this survey.

GENERAL INFORMATION

Contact details

LEGISLATION regarding family approach for potential organ donation

1. Please specify the consent policy for deceased organ donation in your country as enshrined in law: *

Please choose **only one** of the following:

- ◯ Presumed consent (opt-out)
- ◯ Informed/explicit consent (opt-in)
- ◯ Mixed system, please, specify briefly:

1a. If you have a **presumed consent (opt-out)** system in your country:

Only answer this question if the following conditions are met:
-------- Scenario 1 --------
Answer was 'Presumed consent (opt-out)' at question '2 [B1]' (1. Please specify the consent policy for deceased organ donation in your country as enshrined in law:)
-------- or Scenario 2 --------
Answer was 'Other' at question '2 [B1]' (1. Please specify the consent policy for deceased organ donation in your country as enshrined in law:)

Please choose **only one** of the following:

- ◯ Organ donation **may occur** even against the wishes of the family if there is no written objection from the deceased to become a donor ("hard opt-out")
- ◯ Organ donation **will not occur** if the family explicitly objects to donation, even if there is no written objection from the deceased to become a donor ("soft opt-out")

1b. If you have an **informed / explicit consent (opt-in)** system in your country:

Only answer this question if the following conditions are met:
-------- Scenario 1 --------
Answer was 'Informed/explicit consent (opt-in)' at question '2 [B1]' (1. Please specify the consent policy for deceased organ donation in your country as enshrined in law:)
-------- or Scenario 2 --------
Answer was 'Other' at question '2 [B1]' (1. Please specify the consent policy for deceased organ donation in your country as enshrined in law:)

Please choose **only one** of the following:

◯ Organ donation **may occur** in the case of family refusal if there is written consent from the deceased explicitly expressing their willingness to donate

◯ Organ donation **will not occur** in the case of family refusal, even if there is written consent from the deceased explicitly expressing their willingness to donate

2. How can residents in your country express their wishes regarding donation?

❗ Check all that apply
Please choose **all** that apply:

☐ Opt-out registry

☐ Opt-in registry

☐ Other option to express consent or refusal to donate. Please specify briefly: :

3. Please identify the type of regulation that governs the protocols / procedures for determining death by neurological criteria:

❗ Check all that apply
Please choose **all** that apply:

☐ Legislation (legally binding)

☐ Guideline / protocol (non-legally binding)

☐ Other. Please specify briefly::

4. Is there any legislation / guideline that specifies <u>when</u> the healthcare professional must/should inform the family <u>about brain death</u>? *

Please choose **only one** of the following:

- ◯ Yes
- ◯ No

4a. If **YES**, please specify the type of document:

Only answer this question if the following conditions are met:
Answer was 'Yes' at question '7 [B4]' (4. Is there any legislation / guideline that specifies when the healthcare professional must/should inform the family about brain death?)

❗ Check all that apply
Please choose **all** that apply:

- ☐ Legislation
- ☐ Guideline
- ☐ Other. Please specify briefly::

[ ]

4b. If **YES**, please specify when the family must/should be informed about brain death according to the aforementioned legislation/guideline:

Only answer this question if the following conditions are met:
Answer was 'Yes' at question '7 [B4]' (4. Is there any legislation / guideline that specifies when the healthcare professional must/should inform the family about brain death?)

❗ Check all that apply
Please choose **all** that apply:

☐ When brain death is a likely outcome but has not occurred yet

☐ When the patient has a clinical condition consistent with brain death, but the diagnosis has not been officially declared.

☐ When the brain death diagnosis has been officially declared.

☐ Other. Please specify briefly: :

5. Is there any legislation/guideline that determines the category of professional who must/should inform the family about brain death? *

Please choose **only one** of the following:

○ Yes

○ No

5a. If **YES**, please specify the type of document:

Only answer this question if the following conditions are met:
Answer was 'Yes' at question '10 [B5]' (5. Is there any legislation/guideline that determines the category of professional who must/should inform the family about brain death?)

❗ Check all that apply
Please choose **all** that apply:

- [ ] Legislation
- [ ] Guideline
- [ ] Other. Please specify briefly: :

5b. If **YES**, please indicate the category of professional who is permitted to inform the family about brain death according to your national legislation / guidelines

Only answer this question if the following conditions are met:
Answer was 'Yes' at question '10 [B5]' (5. Is there any legislation/guideline that determines the category of professional who must/should inform the family about brain death?)

❗ Check all that apply
Please choose **all** that apply:

- [ ] Medical doctor
- [ ] Nurse
- [ ] Donor co-ordinator
- [ ] Other, please specify: :

6. Is there any legislation / guideline that specifies <u>when</u> the family must/should be approached <u>to address the possibility of organ donation?</u> *

Please choose **only one** of the following:

- ◯ Yes
- ◯ No

6a. If **YES**, please specify the type of document:

Only answer this question if the following conditions are met:
Answer was 'Yes' at question '13 [B6]' (6. Is there any legislation / guideline that specifies when the family must/should be approached to address the possibility of organ donation?)

❶ Check all that apply
Please choose **all** that apply:

- ☐ Legislation
- ☐ Guideline
- ☐ Other. Please specify briefly::

6b. If **YES**, please specify when the family must/should be approached to address the possibility of organ donation according to the aforementioned legislation/guideline

Only answer this question if the following conditions are met:
Answer was 'Yes' at question '13 [B6]' (6. Is there any legislation / guideline that specifies when the family must/should be approached to address the possibility of organ donation?)

❶ Check all that apply
Please choose **all** that apply:

- ☐ When brain death is a likely outcome but has not occurred yet
- ☐ when the patient has a clinical condition consistent with brain death, but the diagnosis has not been officially declared.
- ☐ When the brain death diagnosis has been officially declared

7. Is there any legislation / guideline that determines the category of professional that must/should address <u>organ donation</u> with the family? *

Please choose **only one** of the following:

- ◯ Yes
- ◯ No

7a. If **YES**, please specify the type of document:

Only answer this question if the following conditions are met:
Answer was 'Yes' at question '16 [B7]' (7. Is there any legislation / guideline that determines the category of professional that must/should address organ donation with the family?)

❗ Check all that apply
Please choose **all** that apply:

- ☐ Legislation
- ☐ Guideline
- ☐ Other. Please specify briefly::

7b. If **YES**, please indicate the category of professional who is permitted to address organ donation with the family <u>according to your natonal legislation / guidelines</u>

Only answer this question if the following conditions are met:
Answer was 'Yes' at question '16 [B7]' (7. Is there any legislation / guideline that determines the category of professional that must/should address organ donation with the family?)

❗ Check all that apply
Please choose **all** that apply:

- [ ] Medical doctor
- [ ] Nurse
- [ ] Donor co-ordinator
- [ ] Other. Please specify briefly::

8. Is there any legislation / guideline that provides recommendations on the content of the conversation about organ donation with relatives? *

Please choose **only one** of the following:

- ◯ Yes
- ◯ No

8a. If **YES**, please specify the type of document:

Only answer this question if the following conditions are met:
Answer was 'Yes' at question '19 [B8]' (8. Is there any legislation / guideline that provides recommendations on the content of the conversation about organ donation with relatives?)

❶ Check all that apply
Please choose **all** that apply:

- [ ] Legislation
- [ ] Guideline
- [ ] Other. Please specify briefly::

8b. If **YES**, please briefly explain the recommendations or the required content

Only answer this question if the following conditions are met:
Answer was 'Yes' at question '19 [B8]' (8. Is there any legislation / guideline that provides recommendations on the content of the conversation about organ donation with relatives?)

Please write your answer here:

9. Is there any legislation / guideline covering the withdrawal of life-sustaining treatments after the diagnosis of death by neurologival criteria when organ donation will not proceed <u>for medical or legal reasons?</u> *

Please choose **only one** of the following:

- ◯ Yes
- ◯ No

9a. If **YES**, please specify the type of document:

Only answer this question if the following conditions are met:
Answer was 'Yes' at question '22 [B9]' (9. Is there any legislation / guideline covering the withdrawal of life-sustaining treatments after the diagnosis of death by neurologival criteria when organ donation will not proceed for medical or legal reasons?)

❗ Check all that apply
Please choose **all** that apply:

- ☐ Legislation
- ☐ Guideline
- ☐ Other. Please specify briefly::

10. Is there any legislation / guideline covering the withdrawal of life-sustaining treatments after the diagnosis of death by neurological criteria when organ donation will not proceed due to family refusal? *

Please choose **only one** of the following:

- ◯ Yes
- ◯ No

10a. If **YES,** please specify the type of document:

Only answer this question if the following conditions are met:
Answer was 'Yes' at question '24 [B10]' (10. Is there any legislation / guideline covering the withdrawal of life-sustaining treatments after the diagnosis of death by neurological criteria when organ donation will not proceed due to family refusal?)

❗ Check all that apply
Please choose **all** that apply:

☐ Legislation
☐ Guideline
☐ Other. Please specify briefly::

Practices regarding family approach for potential organ donation

WHERE ARE FAMILIES APPROACHED?

11. Please identify the most common location(s) used to approach the family regarding organ donation in your country

❗ Check all that apply
Please choose **all** that apply:

☐ In the emergency room (ER) in a separate room
☐ In the ER at the patient's bedside
☐ In the intensive care unit (ICU) in a separate room
☐ In the ICU at the patient's bedside
☐ In a dedicated meeting room for relatives within the hospital
☐ Other. Please specify briefly::

WHEN AND HOW ARE FAMILIES APPROACHED?

12. Which is the most common method of providing information to the family about death and organ donation?

❶ Choose one of the following answers
Please choose **only one** of the following:

◯ The information for the family is **always given in one step**, addressing both brain death and organ donation.

(if you choose this option, ***please skip Q14****)*

◯ Normally, the information given is based on when the family is able to process the situation. Depending on their reaction, it may be performed in one step, first addressing brain death, and continuing with organ donation if this information has been understood and they are ready to discuss organ donation.

◯ The information for the family is **always given separately** (decoupling). First, the family is informed about brain death and then later, in a separate interview, **the subject of organ donation is raised**.

◯ Other. Please specify briefly:

13. Please identify when the healthcare professional usually informs the family about brain death:

❶ Check all that apply
Please choose **all** that apply:

☐ When brain death is a likely outcome but has not occurred yet

☐ When the patient has a clinical condition consistent with brain death, but the diagnosis has not been officially declared

☐ When the brain death diagnosis has been officially declared

☐ Other. Please specify briefly::

14. Please identify when the healthcare professional usually informs the family about organ donation (if this meeting is separated from the information about death - ***see Q12***)

Only answer this question if the following conditions are met:
Answer was '
Normally, the information given is based on when the family is able to process the situation. Depending on their reaction, it may be performed in <u>one step</u>, first addressing brain death, and continuing with organ donation if this information has been understood and they are ready to discuss organ donation.

' *or* 'The information for the family is **always given separately** (decoupling). First, the family is informed about brain death and then later, in a separate interview, **the subject of organ donation is raised**.' *or* 'Other' at question '27 [C12]' (WHEN AND HOW ARE FAMILIES APPROACHED? 12. Which is the most common method of providing information to the family about death and organ donation?)

❗ Check all that apply
Please choose **all** that apply:

- ☐ When brain death is a likely outcome but has not occurred yet
- ☐ When the patient has a clinical condition consistent with brain death, but the diagnosis has not been officially declared
- ☐ When the rain death diagnosis has been officially declared
- ☐ Other. Please specify briefly::

WHO HANDLES THE CONVERSATIONS?

15. Which category of healthcare professional <u>usually informs</u> the family <u>about brain death</u>?

❗ Check all that apply
Please choose **all** that apply:

- ☐ Medical doctor
- ☐ Nurse
- ☐ Donor co-ordinator
- ☐ Other, please specify: :

16. Which category of healthcare professional <u>usually informs</u> the family <u>about organ donation</u> in your country?

❗ Check all that apply
Please choose **all** that apply:

- [ ] Medical doctor
- [ ] Nurse
- [ ] Donor co-ordinator
- [ ] Other, please specify: :

17. Is there a training programme on family approach for organ donation in your country? *

Please choose **only one** of the following:

- ◯ Yes
- ◯ No

17a. If **YES**, please specify the target group of healthcare professionals of such a training programme

Only answer this question if the following conditions are met:
Answer was 'Yes' at question '32 [C17]' (17. Is there a training programme on family approach for organ donation in your country?)

❗ Check all that apply
Please choose **all** that apply:

- [ ] Medical doctor
- [ ] Nurse
- [ ] Donor co-ordinator
- [ ] Other, please specify: :

17b. If **YES**, please specify the following:

Only answer this question if the following conditions are met:
Answer was 'Yes' at question '32 [C17]' (17. Is there a training programme on family approach for organ donation in your country?)

Information on family refusal / consent rate for organ donation

18. How many family interviews about DNDD took place in your country in each of the following years?

(i.e. where a healthcare professional talked to the family about DNDD)

❗ Only numbers may be entered in these fields.

19. Please indicate the number of deceased organ donors following diagnosis of death by neurological criteria in your country disaggregated by gender

❗ Only numbers may be entered in these fields.

	female	male
2016:		
2017:		
2018:		
2019:		
2020:		

20. Please indicate the number of potential donors (disaggregated by gender) where DNDD did not take placer (after diagnosis of death by neurological criteria) <u>due to explicit family refusal:</u>

❗ Only numbers may be entered in these fields.

	female	male
2016:		
2017:		
2018:		
2019:		
2020:		

21. Please indicate the number of <u>family refusals</u> for <u>DNDD</u> (before diagnosis of death by neurological criteria had been officially declared) in your country in each of the following years:

❗ Only numbers may be entered in these fields.

22. Please indicate the number of <u>initial family refusals</u> for DNDD in your country in each of the following years:

(i.e. where the relatives of the potential organ donor expressed their refusal for organ donation at any time during the conversation(s), regardless of whether organ donation ultimately took place)

NOTE: Reversal of DNDD refusals = no. of initial DNDD family refusals - no. of DNDD family refusals

❗ Only numbers may be entered in these fields.

DCDD SECTION. This section covers donation after circulatory determination of death

23. Is DCDD permitted and practised in your country? *

Please choose **only one** of the following:

◯ Yes

◯ No

If you answer "No" to this question, you will be redirected to question 31.

24. Please indicate the number of family interviews about DCDD (i.e. where a healthcare professional talked to the relatives about DCDD) **that took place in your country in each of the following years:**

Only answer this question if the following conditions are met:
Answer was 'Yes' at question '40 [E23]' (23. Is DCDD permitted and practised in your country?)

❗ Only numbers may be entered in these fields.

25. Please indicate the number of deceased organ donors following diagnosis of death by circulatory criteria in your country disaggregated by gender

Only answer this question if the following conditions are met:
Answer was 'Yes' at question '40 [E23]' (23. Is DCDD permitted and practised in your country?)

Only numbers may be entered in these fields.

	female	male
2016:		
2017:		
2018:		
2019:		
2020:		

26. Please indicate the number of <u>family refusals</u> for DCDD (disaggregated by gender) where organ donation did not proceed due to family refusal in your country in each of the following years:

Only answer this question if the following conditions are met:
Answer was 'Yes' at question '40 [E23]' (23. Is DCDD permitted and practised in your country?)

❗ Only numbers may be entered in these fields.

	female	male
2016:		
2017:		
2018:		
2019:		
2020:		

27. Please indicate the number of initial family refusals for DCDD in your country in each of the following years:

(i.e. where the relatives of the potential organ donor expressed their refusal for DCDD at any time durng the conversation(s), regardless of whether organ donation ultimately took place)

NOTE: Reversal of DCDD refusals - no. of initial DCDD family refusals - No. of DCDD family refusals

Only answer this question if the following conditions are met:
Answer was 'Yes' at question '40 [E23]' (23. Is DCDD permitted and practised in your country?)

❗ Only numbers may be entered in these fields.

28. When relatives are approached for their consent for organ donation prior to the determination of death by neurological criteria, are the two possible types of donation (DNDD / DCDD) discussed with them separately?

Only answer this question if the following conditions are met:
Answer was 'Yes' at question '40 [E23]' (23. Is DCDD permitted and practised in your country?)

❗ Choose one of the following answers
Please choose **only one** of the following:

- ◯ Donation is discussed without providing specific details
- ◯ Both types of donation are explained and discussed in every interview
- ◯ Both types of donation are explained and discussed in every interview

29. Is it normal practice in your country to switch occasionally from DNDD to DCDD? *

Only answer this question if the following conditions are met:
Answer was 'Yes' at question '40 [E23]' (23. Is DCDD permitted and practised in your country?)

Please choose **only one** of the following:

- ◯ Yes
- ◯ No

29a. If **YES**, please provide the number of cases in 2020:

Only answer this question if the following conditions are met:
Answer was 'Yes' at question '46 [E29]' (29. Is it normal practice in your country to switch occasionally from DNDD to DCDD?)

❗ Only numbers may be entered in this field.
Please write your answer here:

30. Is it normal practice in your country to switch occasionally from DCDD to DNDD? *

Only answer this question if the following conditions are met:
Answer was 'Yes' at question '40 [E23]' (23. Is DCDD permitted and practised in your country?)

Please choose **only one** of the following:

- ◯ Yes
- ◯ No

30a. If **YES**, please provide the number of cases in 2020:

Only answer this question if the following conditions are met:
Answer was 'Yes' at question '48 [E30]' (30. Is it normal practice in your country to switch occasionally from DCDD to DNDD?)

❗ Only numbers may be entered in this field.
Please write your answer here:

Legislation and practices for organ donation from foreign / non-resident deceased persons

31. Is there any legislation / guideline in your country that addresses the possibility of donation from a foreign / non-resident deceased person? *

Please choose **only one** of the following:

- ◯ Yes
- ◯ No

31a. If **YES**, which of the following applies?:

Only answer this question if the following conditions are met:
Answer was 'Yes' at question '50 [F31]' (31. Is there any legislation / guideline in your country that addresses the possibility of donation from a foreign / non-resident deceased person?)

Please choose **all** that apply:

- [ ] Legislation (legally-binding)
- [ ] Guideline / Protocol (non-legally binding)

31b. If **YES**, please choose one of the two options below:

Only answer this question if the following conditions are met:
Answer was 'Yes' at question '50 [F31]' (31. Is there any legislation / guideline in your country that addresses the possibility of donation from a foreign / non-resident deceased person?)

❗ Choose one of the following answers
Please choose **only one** of the following:

- ○ Organ donation from foreign / non-resident deceased persons **is possible** in my country
- ○ Organ donation from foreign / non-resident deceased persons **is not permitted** in my country

31c. If **YES**, please briefly describe the content of the legislation / guideline:

Only answer this question if the following conditions are met:
Answer was 'Yes' at question '50 [F31]' (31. Is there any legislation / guideline in your country that addresses the possibility of donation from a foreign / non-resident deceased person?)

Please write your answer here:

31d. If **NO**, please briefly describe the applied practice:

Only answer this question if the following conditions are met:
Answer was 'No' at question '50 [F31]' (31. Is there any legislation / guideline in your country that addresses the possibility of donation from a foreign / non-resident deceased person?)

Please write your answer here:

32. According to the regulation / practices applied covering donation from non-residents:

❗ Choose one of the following answers
Please choose **only one** of the following:

◯ There is no difference in the process of organ donation if the deceased is foreign / non-resident compared to a resident donor. The law is applied as for any other donor where the deceased is admitted to hospital

◯ The law of the country of origin of the deceased must be applied if they are foreign / non-resident, irrespective of the country where the donor is admitted

33. As regards the approach to the family of a foreign / non-resident deceased donor:

❗ Choose one of the following answers
Please choose **only one** of the following:

◯ The family is contacted *(via a close contact, the embassy of the country of origin, police, or any other means)* and approached about organ donation, irrespective of the national consent system in place in the country where the donor is admitted to hospital

◯ The family's wishes are never taken into consideration in the case of organ donation.

Submit your survey.
Thank you for completing this survey.
